# Changes in sodium levels in Australian packaged foods between 2014 and 2019: an interrupted time series analysis of the impact of the Victorian Salt Reduction Partnership’s media advocacy strategy

**DOI:** 10.1186/s12966-023-01475-5

**Published:** 2023-06-14

**Authors:** Emalie Rosewarne, Joseph Alvin Santos, Gian Luca Di Tanna, Maria Shahid, Carley Grimes, Kristy A. Bolton, Jacqui Webster, Bruce Neal, Mark Woodward, Daisy Coyle, Kathy Trieu

**Affiliations:** 1 The George Institute for Global Health, The University of New South Wales, Sydney, NSW Australia; 2 Institute for Physical Activity and Nutrition, School of Exercise and Nutrition Sciences, Deakin University, Geelong, Australia; 3 The George Institute for Global Health, School of Public Health, Imperial College London, London, UK

**Keywords:** Sodium, Salt, Nutrition, Public health, Food policy, Dietary intervention, Hypertension

## Abstract

**Background:**

The Victorian Salt Reduction Partnership (VSRP) implemented a media advocacy strategy (intervention) to stimulate food manufacturers to reduce sodium levels across targeted Australian packaged foods between 2017 and 2019. This study assessed changes in sodium levels of targeted and non-targeted packaged foods during the intervention (2017 to 2019) compared to before the intervention (2014 to 2016) in Australia.

**Methods:**

Annually collected branded-food composition data from 2014 to 2019 were used. Interrupted time series analyses was conducted to compare the trend in sodium levels in packaged foods during the intervention (2017–2019) to the trend in the pre-intervention period (2014–2016). The difference between these trends was derived to estimate the effect of the intervention.

**Results:**

A total of 90,807 products were included in the analysis, of which 14,743 were targeted by the intervention. The difference in before and during intervention trends between targeted and non-targeted food categories was 2.59 mg/100 g (95% CI: -13.88 to 19.06). There was a difference in the pre-intervention slope (2014, 2015, 2016) and intervention slope (2017, 2018, 2019) for four of 17 targeted food categories. There was a decrease in sodium levels (mg/100 g) in one food category: frozen ready meals (-13.47; 95% CI: -25.40 to -1.53), and an increase in three categories: flat bread (20.46; 95% CI: 9.11 to 31.81), plain dry biscuits (24.53; 95% CI: 5.87 to 43.19), and bacon (44.54; 95% CI: 6.36 to 82.72). For the other 13 targeted categories, the difference in slopes crossed the line of null effect.

**Conclusions:**

The VSRP’s media advocacy strategy did not result in a meaningful reduction in sodium levels of targeted packaged food products during the intervention years compared to trends in sodium levels before the intervention. Our study suggests media advocacy activities highlighting the differences in sodium levels in packaged food products and industry meetings alone are not sufficient to lower average sodium levels in packaged foods in the absence of government leadership and measurable sodium targets.

**Supplementary Information:**

The online version contains supplementary material available at 10.1186/s12966-023-01475-5.

## Background

Australians are consuming almost double the recommended amount of dietary sodium, with a current best estimate of 3,840 mg per day (9.6 g salt) [1] compared to the recommendation of less than 2,000 mg per day (5 g salt) [1, 2], leading to raised blood pressure and an increased risk of cardiovascular diseases [3]. Sodium intake is primarily from processed and packaged foods in Australia [4, 5]. As such, reducing sodium levels in the Australian packaged food supply, particularly by targeting the top sources of dietary sodium and high sodium foods, is a key population health strategy.

In 2014, the Federal Government of Australia was taking minimal action towards reducing population sodium intake [6]. The Government’s Food and Health Dialogue sodium reformulation program (initiated in 2009) had lapsed the year prior [7], and two new voluntary initiatives were being proposed: The Healthy Food Partnership sodium reformulation targets and Health Star Rating front-of-pack nutrition labelling system [6]. However, for these proposed initiatives, the timeframe for implementation was not yet known, no mechanisms to promote uptake or support industry action were developed, and no plans were established to monitor and evaluate progress [6].

During the absence of any national level action to reduce sodium in the packaged food supply, the Victorian Salt Reduction Partnership (hereon referred to as the VSRP) – a state-based public health partnership – was established in 2014 to address the gap through a 5-year multi-faceted sodium reduction strategy. The VSRP was led by the Victorian Health Promotion Foundation and comprised government, non-government and research organisations [8]. The VSRP aimed to reduce average population sodium intake in the state of Victoria by 1 g per day by 2020 [9] through a four-armed strategy consisting of consumer awareness, generating public debate, food industry engagement, and policy and advocacy strengthening [8].

The *generating public debate* arm was intended to be a central lever to support the achievement of consumer awareness, food industry and policy outcomes [8]. The mechanism to achieve this was a media advocacy strategy (implemented between 2017 and 2019), called *Unpack the Salt,* based on a similar intervention undertaken in the UK [10]. The strategy aimed to 1) engage food manufacturers in conversations about sodium reduction reformulation and support them to take action to reduce sodium levels in their packaged food products, 2) inform consumers about the high variability of sodium in similar foods and encourage them to select the lower sodium alternatives, and 3) advocate for sodium reduction policy change at state and federal levels of government, including government-led sodium reduction targets [11, 12].

Six reports covering 17 targeted product categories were produced by researchers that showed the variability in sodium levels of different products within each of the categories, and named the brands and products with the highest and lowest sodium levels per 100 g [11, 13]. The key findings of reports were translated into media releases, which were periodically disseminated by VSRP organisations through mass and social media (e.g. television, radio, newspapers, twitter, and online news) [11]. Food manufacturers with the highest sodium products within their category were contacted by the VSRP prior to the media release to notify them, allow them to prepare a response, and offer them an opportunity to meet to discuss reformulation and how the VSRP could support them to do this [11].

**Fig. 1:**
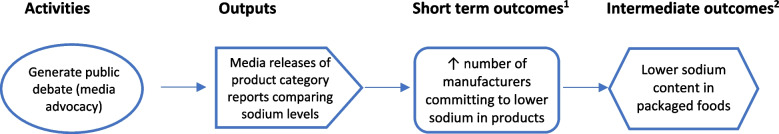
Excerpt adapted from the revised logic model of the Partnership program from Rosewarne et al. [8]. Reproduced with permission. ^1^Indicators of media coverage and industry engagement from the media advocacy strategy have been previously published [11]. ^2^This study aims to assess whether sodium levels in packaged foods were reduced as a result of the media advocacy strategy

Our interim process evaluation of the VSRP’s media advocacy strategy assessed the extent of media coverage and industry engagement achieved. The media advocacy strategy reached between 2.3 and 7.5 million Australian consumers per media release. Additionally, a total of 10 food manufacturers were engaged in meetings about reducing sodium levels in their packaged food products. Most manufacturers had two or more meetings but few took up proposed activities to support reformulation (e.g. producing a sodium reformulation case study, benchmarking sodium levels against other similar products) [11]. The 10 manufacturers engaged included Australia’s four major retailers, three large manufacturers and three smaller manufacturers [11], together estimated to contribute to more than 45% of Australian’s sodium purchases [14]. While the media advocacy strategy achieved high reach among the general population and engagement with food manufacturers responsible for almost half of all sodium purchases in Australia [11], it was unclear to what extent the media advocacy strategy resulted in changes to sodium levels in the Australian packaged food supply (Fig. 1).

Therefore, this study aimed to assess changes in sodium levels in the Australian packaged food supply as a result of the VSRP’s media advocacy strategy, by comparing changes in sodium levels of targeted products and non-targeted products before the intervention (2014 to 2016) and during the intervention (2017 to 2019). The secondary aim was to assess trends in sodium levels of different categories of targeted packaged foods during the intervention compared to before the intervention.

## Methods

### Data source

This study utilised The George Institute for Global Health’s Australian FoodSwitch Database: a database containing information on the nutritional composition of packaged foods available in Australia from annual data collections in four major supermarkets. Together, these four supermarkets have almost 85% market share [15], and each year, data on more than 20,000 products are collected using a purpose-built Data Collector App. Data collectors visit supermarkets and take a series of photographs of food item packaging and nutrition information. This process is described in more detail in Dunford et al. [16].

### Data inclusion and exclusion

FoodSwitch data from annual data collections from 2014 to 2019 were included in this study. Data extracted from the database included manufacturer, brand and product name, package size (g), sodium level (mg/100 g), and food category and subcategory name.

**Fig. 2:**
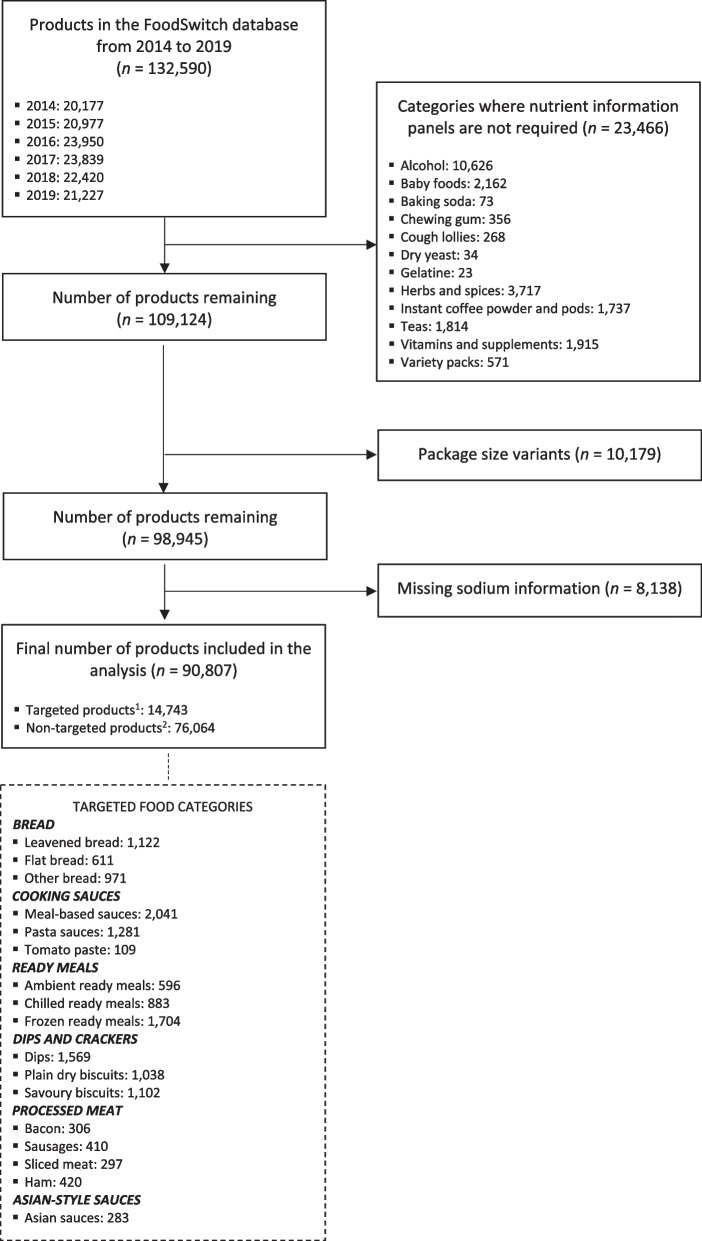
The number of products included in the analysis and product exclusions. ^1^Targeted products were products within 17 food categories targeted in the six reports produced by the Victorian Salt Reduction Partnership. ^2^Non-targeted products were all remaining food products

Food category and product exclusions are outlined in Fig. 2. Food categories where manufacturers are not required to display a nutrient information panel were excluded [17]. For products where the same formulation was sold in different package sizes (e.g. a big and small bottle of soy sauce), only one instance of the product was included to avoid double counting. These package size variants were identified by the same product name and sodium level despite different package sizes. Products missing sodium information because they are not required to display a nutrition information panel (e.g. fresh bread, produce or meat) or where the nutrition information panel contains sodium levels as prepared by consumers (rather than sodium as sold on shelves) were also excluded.

Included food products were then categorised as targeted or non-targeted (control). Targeted products were those covered by the six reports disseminated through mass and social media in 2017 and 2018 as part of the VSRP’s media advocacy strategy [11]. The reports were on sodium levels in bread, cooking sauces, ready meals, dips and crackers, processed meat, and Asian-style sauces, which were chosen as they were high contributors to population sodium intake or high sodium products [11]. The foods from the six reports were broken down into 17 targeted product categories for the analysis as there was large variation in the types of products included in each report. Non-targeted (or control) products were all remaining food products (not excluded in earlier steps). For the main analysis, targeted and non-targeted products were included so that we could understand whether sodium levels across the food supply changed due to or regardless of the intervention; for the secondary analysis, only targeted products were included to see whether the intervention had had an impact on the sodium levels of specific categories of packaged foods (Fig. 2).

### Data analysis

An interrupted time series analysis was conducted to compare the trend in sodium levels in foods during the media advocacy intervention (2017–2019) to the trend in the pre-intervention period (2014–2016) [18–20]. Individual-level data (instead of aggregate-level data, which is commonly used in standard segmented regression analysis [20]) was used, and mixed-effects linear regression models were fitted by Restricted Maximum Likelihood to take into account product clustering (i.e. the matched nature of some of the food products over time) [21]. The years of data collection were divided into pre-intervention (2014, 2015, and 2016) and intervention (2017, 2018, and 2019) segments, and separate slopes were estimated in each segment. The difference between these slopes was derived to estimate the effect of the intervention.

For the primary analysis, the interrupted time series model for multiple group analysis was used [18–20]:

$$Y= {\beta }_{0}+ {\beta }_{1}T+ {\beta }_{2}X+{\beta }_{3}XT+ {\beta }_{4}G+ {\beta }_{5}GT+ {\beta }_{6}GX+{\beta }_{7}GXT$$

In this equation, $Y$ is the outcome variable (i.e. sodium levels in foods in mg/100 g), $T$ is the time since the start of the study (i.e. 2014 is 1, 2015 is 2, 2016 is 3 etc.), $X$ is the level of the intervention (i.e. pre-intervention is 0, intervention is 1), $G$ is the group (i.e. non-targeted categories are 0 and targeted categories are 1) and $XT$ is the interaction between time and the level of intervention.

The regression output provides the intercept for non-targeted food categories (${\beta }_{0}$), pre-intervention trend for non-targeted food categories (${\beta }_{1}$), the change in the level of outcome of non-targeted food categories at the time of intervention (${\beta }_{2}$), the difference between the pre-intervention and intervention trends for non-targeted food categories (${\beta }_{3}$), the difference between slopes for targeted and non-targeted food categories in the pre-intervention period (${\beta }_{5}$), the difference between change in the level of outcome at the time of intervention for targeted and non-targeted food categories (${\beta }_{6}$), and the difference between slopes for targeted and non-targeted food categories in the intervention period (${\beta }_{7}$). The lincom command was used to calculate the pre-intervention trend for targeted food categories (${\beta }_{1}$ + ${\beta }_{5}$), intervention trend for targeted food categories (${\beta }_{1}$ + ${\beta }_{3}+ {\beta }_{5}$ + ${\beta }_{7}$), intervention trend for non-targeted food categories (${\beta }_{1}$ + ${\beta }_{3}$), difference between the targeted and non-targeted food categories trends in the intervention period (${\beta }_{5}$ + ${\beta }_{7}$), and difference in targeted food categories pre-intervention and intervention trends (${\beta }_{3}$ + ${\beta }_{7}$); which are not part of the default regression output.

For the secondary analysis of targeted food categories, the standard form of interrupted time series model was used [18–20]:

$$Y= {\beta }_{0}+ {\beta }_{1}T+ {\beta }_{2}X+{\beta }_{3}XT$$

In this equation, $Y$ is the outcome variable (i.e. sodium levels in foods in mg/100 g), $T$ is the time since the start of the study (i.e. 2014 is year 1, 2015 is year 2, 2016 is year 3 etc.), $X$ is the level of the intervention (i.e. pre-intervention is 0, intervention is 1) and $XT$ is the interaction between time and the level of intervention. By default, the regression output provides the intercept (${\beta }_{0}$), the pre-intervention trend (${\beta }_{1}$), the *level change* or the immediate change in the level of outcome following the start of the intervention (${\beta }_{2}$), and the difference between the pre-intervention and intervention trends (${\beta }_{3}$). The post-intervention trend (${\beta }_{1}$ + ${\beta }_{3}$) was derived post-estimation. In this analysis, the difference between the pre-intervention and intervention trends was treated as the primary outcome measure, but the separate slopes pre-intervention, intervention, and level change were also examined to help explain the observed differences. For the secondary analysis comparing targeted and non-targeted food categories, the regression model suggested by Linden et al. for multiple-group analysis was followed [18, 19].

Figures were generated for each product category to illustrate the effect of the intervention. To check for the presence of systematic over- or under- estimation due to mixed effects modelling, the observed values (raw mean sodium levels per year) were calculated and plotted against the predicted values (from the interrupted time series analysis model). Boxplots were also added to show the observed distribution of sodium levels in foods across the years. All analyses were conducted in Stata version 15.1 for Windows (StataCorp LLC, TX, USA). Figures were generated in Stata and R version 1.2.1572 [22].

## Results

A total of 90,807 products were included in the analysis, after applying category exclusions (*n* = 23,466), duplicate product exclusions (*n* = 10,179) and missing sodium exclusions (*n* = 8,138). Of these, 14,743 products (16%) from 17 food categories were targeted by the VSRP’s media advocacy strategy (Fig. 2). The number of products within each food category ranged from 109 (in tomato paste products) to 2,041 products (in meal-based sauces).

### Comparison of changes in sodium levels between targeted and non-targeted packaged food products

**Fig. 3:**
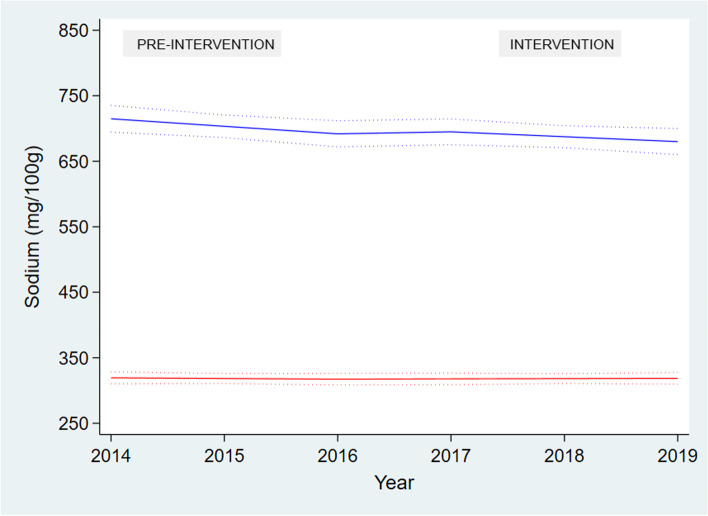
The pre-intervention and intervention slopes for targeted and non-targeted foods. Solid line represents the linear prediction calculated using interrupted time series analysis; dashed lines around the solid line represents the 95% confidence intervals of the linear prediction. Blue line represents targeted foods; red line indicates non-targeted foods

**Fig. 4:**
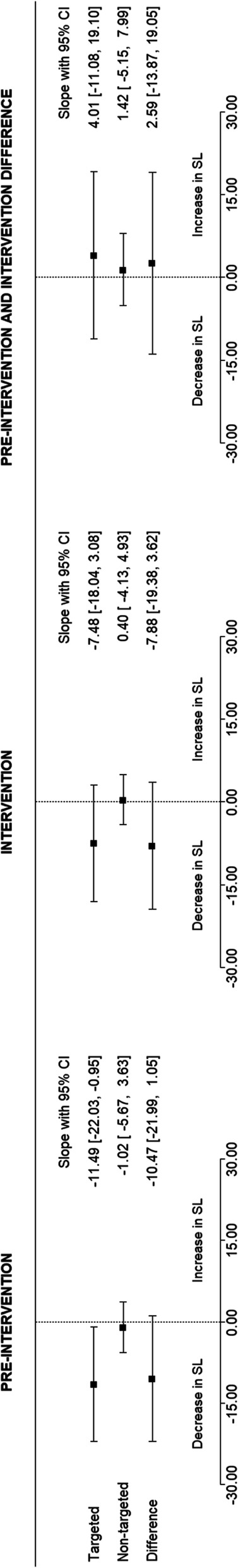
The differences between pre-intervention (2014–2016) and intervention (2017–2019) slopes for targeted and non-targeted foods, calculated using interrupted time series analysis, with 95% confidence intervals. SL: Sodium levels, mg/100 g

Figure 3 illustrates the pre-intervention and intervention slopes for targeted and non-targeted foods. Overall, there was no difference in the trends from the pre-intervention period to the intervention period between targeted and non-targeted products (2.59 mg/100 g; 95% CI: -13.88 to 19.06) (Fig. 4). However, there was a decrease in sodium levels among targeted foods (-11.49 mg/100 g; 95% CI: -22.03 to -0.95) during the pre-intervention period only, while all other slopes crossed the line of null effect (Fig. 4).

### Effect of the intervention on sodium levels of each targeted food category.

**Fig. 5:**
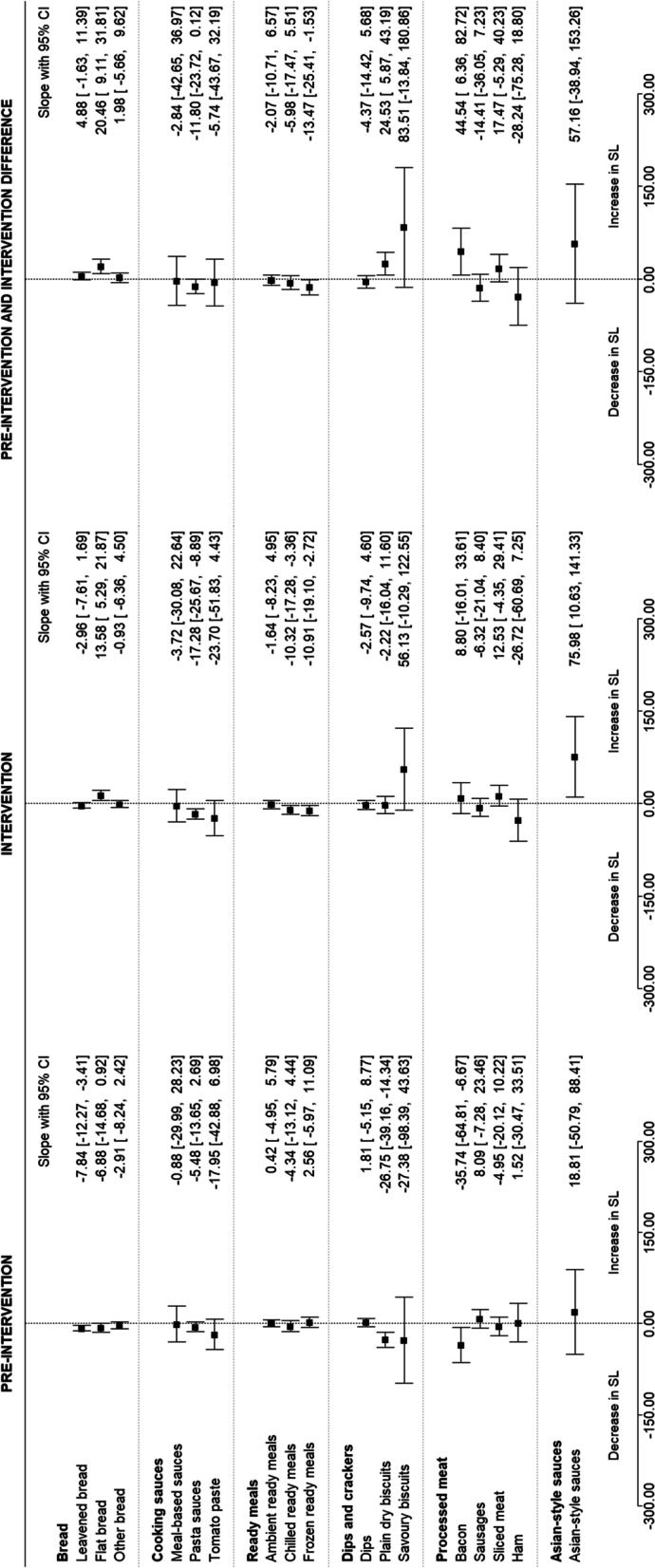
The estimated differences between pre-intervention (2014–2016) and intervention (2017–2019) slopes for targeted food categories, including estimated change in sodium levels in the targeted food categories before and during the intervention, calculated using interrupted time series analysis, with 95% confidence intervals. SL: Sodium levels, mg/100 g

For four of the 17 targeted food categories examined, the change in sodium levels during the intervention period (2017–2019) was different to the change during the pre-intervention period (2014–2016) (Fig. 5). Comparing the pre-intervention and intervention slopes showed a decrease in sodium levels in one category: frozen ready meals (-13.47 mg/100 g; 95% CI: -25.40 to -1.53); and an increase in three categories: flat bread (20.46 mg/100 g; 95% CI: 9.11 to 31.81), plain dry biscuits (24.53 mg/100 g; 95% CI: 5.87 to 43.19), and bacon (44.54 mg/100 g; 95% CI: 6.36 to 82.72). For the other 13 targeted food categories, the difference in slopes crossed the line of null effect (Fig. 5).

**Fig. 6:**
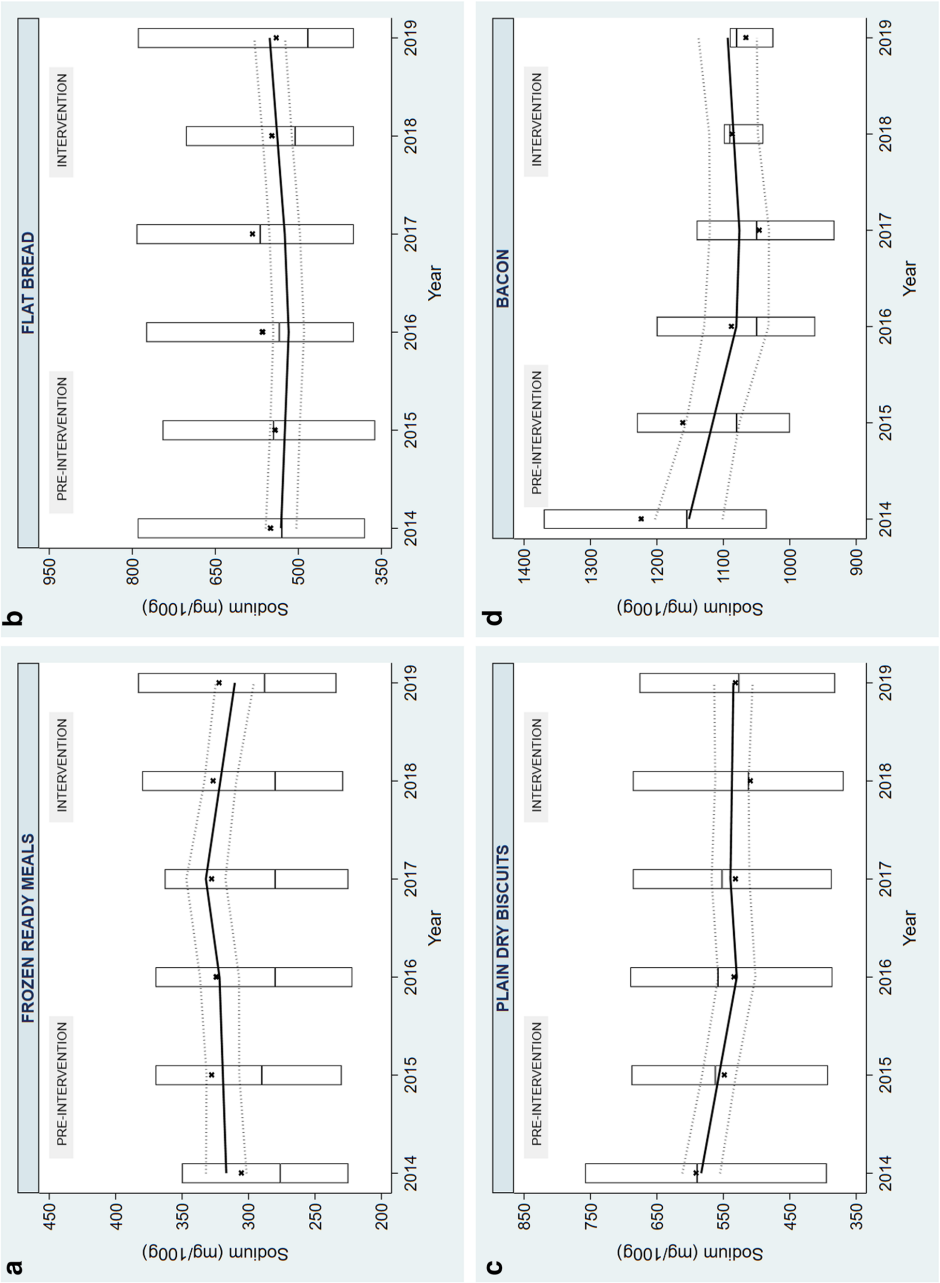
Trends for the food categories where there was a difference in pre-intervention and intervention slopes. Black dot represents the raw mean (Additional File 1: Supplementary Table 2); solid line represents the linear prediction; dashed lines around the solid line represents the 95% CI of the linear prediction, the box plot shows the median and interquartile range

The trends during the pre-intervention period (2014–2016), the intervention period (2017–2019) and the period between these two (2016–2017) were also separately examined (Additional File 1: Supplementary Table 1) and can explain the net differences in the pre-intervention and intervention slopes found in the above four categories (Fig. 6). For frozen ready meals, there was a decrease in sodium levels during the intervention period (-10.91 mg/100 g; 95% CI: -19.10 to -2.72) compared to no change pre-intervention (Fig. 6). For flat bread, there was an increase in sodium levels during the intervention period (13.58 mg/100 g; 95% CI: 5.29 to 21.87) compared to no change pre-intervention. For plain dry biscuits and bacon, there was a decrease in sodium levels prior to the intervention (plain dry biscuits: -26.75 mg/100 g; 95% CI: -39.16 to -14.34 and bacon: -35.74 mg/100 g; 95% CI: -64.80 to -6.67), followed by no change in sodium levels during the intervention period (Fig. 6). When only examining the intervention period (2017–2019), there was a decrease in sodium levels in three categories (pasta sauces, chilled ready meals, and frozen ready meals) and an increase in two categories (flat bread and Asian-style sauces; Fig. 5). During the pre-intervention (2014–2016), there was a decrease in sodium levels in three product categories (leavened bread, plain dry biscuits and bacon) (Fig. 5). Between the pre-intervention and intervention periods (2016 and 2017; level change), there was an increase in three categories (chilled ready meals, frozen ready meals and sausages;  Additional File 1: Supplementary Table 1). The separate changes for each of the periods (pre-intervention slope, level change and intervention slopes) for the other food categories crossed the line of null effect (Additional File 1: Supplementary Table 1).

## Discussion

This analysis showed that there were no meaningful differences in the trends from the pre-intervention and post-intervention period between the Partnership-targeted and non-targeted food products. Minor differences in pre-post intervention trends in sodium levels were observed for four of 17 targeted food categories, although only one of these—frozen ready meals—was a reduction in sodium levels. This study is unique in using longitudinal data from a comprehensive brand-specific food composition database collected annually to evaluate changes in sodium levels across the entire Australian packaged food supply and across several years.

### Comparison to successful strategies to reduce sodium in packaged foods

The VSRP media advocacy strategy did not result in a meaningful change in sodium levels across targeted packaged foods with null effect demonstrated overall and for 13 of 17 targeted food categories. Further, changes in sodium levels in the four categories where there was a difference between the pre-intervention and intervention slopes are unlikely to be related to the media advocacy intervention. These findings are unlike the similar approach led by UK expert group Action on Salt that the VSRP intervention was based on [10]. An important difference is that the UK media advocacy strategy was supplemented by the establishment of the UK government’s first sodium reformulation targets in 2006, which applied to 85 food categories that contribute to population sodium intake [10]. This combined approach successfully stimulated salt reduction through reformulation action for selected food categories by food manufacturers between 2004 and 2011 [10]. Although, reductions varied from 14 to 57% across food categories [10]. This approach continues to be implemented in the UK, with sodium reformulation targets set to be achieved by 2024 by the UK government [23] and regular media advocacy led by Action on Salt [24]. However, more recent evidence indicates the UK strategy may no longer be having the desired impact on sodium levels in the food supply [25], and the latest data illustrates large variations in compliance of different product categories with the targets [26]. It is likely that Government leadership in establishing sodium reformulation targets was the main catalyst for initial food manufacturer action, but as progressively lower sodium targets have been set with no motivation for manufacturers to comply (e.g. consequences for non-compliance or threat of legislation), action has stagnated [27]. Such findings suggest successful sodium reduction in packaged foods in Australia will require a comprehensive approach to sodium reduction including a strong media strategy alongside government-led targets with robust monitoring of compliance.

Many other countries around the world are working with the food industry to reduce sodium levels in the packaged food supply through food reformulation strategies [28, 29]; but no identified approach was similar to the VSRP’s media advocacy strategy. Most countries (*n* = 43/62) with reformulation strategies have established voluntary or mandatory sodium reformulation targets for packaged foods, alone or in combination with other industry engagement approaches [29]. The remaining 19 countries have not set sodium targets and are attempting to reduce sodium levels in the food supply through industry engagement strategies, such as voluntary agreements between government and food companies, and industry meetings [29]. Of the 19, only three countries have evaluated the impact of their industry engagement strategies (where sodium targets were not established) on sodium levels in the food supply [29]. These three countries reported reductions in sodium levels as a result of voluntary agreements between the Ministry of Health and specific food manufacturers or associations to reduce sodium levels, however the robustness of these findings are uncertain [29]. In Morocco, voluntary agreements between the Ministry of Health and the national bakery and pastry federation has reportedly resulted in sodium reductions in bread of 26% [30]. Italy reported that voluntary agreements between the Ministry of Health and national associations of bakers, pasta manufacturers, food product industries have resulted in sodium reductions in bread, gnocchi, selected ready meals and soups of 10 to 15% [31]. Mongolia reported that voluntary agreements with manufacturers of bread, processed meats, soups and sauces have resulted in sodium reductions by participating manufacturers of almost 30% in these food categories [29]. These findings suggest voluntary agreements between government agencies and food manufacturer associations may be an effective strategy for reducing sodium levels in some food categories, particularly in contexts where there are just a few market-dominant manufacturers. While the VSRP’s media advocacy strategy resulted in meetings with 10 major food companies who had a combined market share of almost 30% [32] and together contributed to more than 45% of Australian’s sodium purchases [14], no voluntary agreements were made between the VSRP and food manufacturers or associations [11]. This may be one explanation for no meaningful changes in sodium levels in targeted packaged foods. Additionally, our study, in line with experiences in other countries, suggests there remains no evidence that meetings with the food industry alone can stimulate reformulation action [29].

Thus, there are two important differences between these successful strategies and the VSRP initiative: government leadership/involvement and measurable goals (e.g. sodium targets or voluntary agreements). These two factors are present when governments establish sodium reformulation targets across the packaged food supply or when government agencies create voluntary agreements with food industry associations/stakeholders to reduce sodium levels in foods. The establishment and implementation of sodium targets, particularly maximum level sodium targets has the added advantage of creating a level playing field for all manufacturers [29, 33]. In Australia, the VSRP’s media advocacy strategy was implemented in the absence of government-led sodium reformulation targets. In fact, the media advocacy activities were executed in 2017 and 2018, between the two Federal Government initiatives that established sodium reformulation targets: the Food and Health Dialogue (targets set for 2009 to 2013) and the Healthy Food Partnership (targets set for 2020 to 2025) [7, 34]. The current study suggests there may have been some effect lag (time between targets being set and manufacturers taking action to lower sodium levels in their foods) from the Food and Health Dialogue observed during the pre-intervention period (2014–2016) for three food categories (leavened bread, plain dry biscuits and bacon), although we did not undertake a formal analysis. A previous study that showed the Food and Health Dialogue targets had resulted in reductions in sodium levels in leavened bread (9%) and selected processed meats (8%; bacon, ham and cured meats) from 2010 to 2013 [35]. We observed continued reductions in sodium levels in leavened bread and bacon, but not ham, during 2014–2016. We also observed reductions in sodium levels in plain dry biscuits, but not savoury crackers or any category of cooking sauces, during the pre-intervention period, all of which were also targeted by the Food and Health Dialogue. However, the sodium reduction effect did not continue beyond 2016 in the absence of sodium reformulation targets. Throughout the intervention, the VSRP advocated for new national sodium targets, however it wasn’t until after the intervention, in 2020, that targets were established by the Healthy Food Partnership [34]. The VSRP was able to influence the sodium targets by providing responses to public and targeted consultations [9], and influence manufacturers to reformulate by developing resources which were disseminated by the Healthy Food Partnership [36]. However, in the absence of government-led targets during the intervention period, implementing a media advocacy did not result in meaningful food supply impact. Interim findings of another advocacy intervention delivered by a non-governmental organisation directly to food manufacturers to reduce the sodium levels in processed foods in Australia also suggested that no corporate nutrition actions to reduce sodium were taken following the advocacy intervention [37]. These findings suggest that in the absence of government leadership and measurable goals (sodium targets or voluntary agreements), media advocacy activities alone cannot generate sufficient drive for manufacturers to reformulate high sodium foods or sustain reduced sodium levels as a result of a previous government program.

### Contribution to the VSRP’s goal in reducing population sodium intake

While the media advocacy strategy could be used to engage food manufacturers in meetings about sodium reformulation [11], this did not translate to reductions in sodium levels in the food supply. Since the majority of Victoria’s sodium consumption is from processed and packaged foods [4], the lack of impact of the VSRP’s media advocacy strategy on lowering sodium levels in the food supply likely means there will be no meaningful reduction in population sodium consumption.

The media advocacy strategy was also supported by three other intervention arms, including a wider food industry engagement strategy. The food industry engagement strategy involved the development of a reformulation guide for food manufacturers [36], a benchmarking service for companies, and innovation grants [8]. However, these activities commenced later in the intervention period [8]; for example, the reformulation guide was not published until mid-2019 [38]. As such, if there is an effect of these additional activities on sodium levels in the packaged food supply, it will likely not be seen until well after the intervention period.

### Implications for policy and practice

Together with evidence from previous studies, our findings suggest that media advocacy strategies resulting in meetings with manufacturers to discuss approaches to reformulation [11] are not enough to stimulate sufficient manufacturer action to have an impact on sodium levels within the food supply when implemented alone. However, media advocacy strategies and industry meetings may help increase compliance with established sodium reformulation targets, as seen in countries such as the UK [10]. Strong government leadership and measurable sodium level goals are two key ingredients likely to be necessary to lower sodium levels across the packaged food supply. The establishment and implementation of stringent sodium reduction targets for packaged foods, such as those proposed in the new World Health Organization sodium benchmarks [39], along with strong governance, monitoring and evaluation, should reduce sodium levels in the packaged food supply.

### Strengths and limitations

The interrupted time series analysis allowed for an assessment of the trends in sodium levels in food categories during the intervention period relative to the trend in the pre-intervention period. This analysis is more robust than comparing one time point before the intervention and one time point after the intervention. By undertaking this approach, we were able to account for changes to sodium levels in the packaged food supply before the intervention and we observed a potential lag effect of the Australian government’s previous sodium reduction initiative. In using individual-level data, we were able to take into account product clustering (i.e. the same food products observed over time). The analysis used data from a comprehensive food composition database covering almost 85% of the Australian market share [15], and more than 90,000 products were able to be included in the study. There are also some limitations that should be considered. Firstly, we were unable to account for the sales volume of different products within each food category, and therefore unable to determine the change in sales-weighted sodium levels. Secondly, there may be a delay in the effect the VSRP intervention as reformulation is a gradual process undertaken by food manufacturers. With media advocacy activities continuing through to early 2020, though food supply data was limited to 2019, the full effect of the VSRP strategy may not have been observed. Future analyses of sodium levels in targeted and non-targeted food categories for up to several years following the intervention should be undertaken to determine any effect of the VSRP strategy.

## Conclusions

The VSRP intervention did not result in any meaningful reduction in sodium levels of packaged food products targeted by the media advocacy strategy. Our study suggests media advocacy and industry engagement strategies alone are unlikely to be effective in stimulating changes in sodium levels in the packaged food supply in the absence of strong government leadership and measurable sodium targets for packaged foods.

## Supplementary Information

**Additional file 1: Supplementary Table 1. **Estimated change in sodium levels in the targeted food products before and during the intervention. **Supplementary Table 2. **The number of products, mean sodium content (mg/100g) and standard deviation for each food category before (2014-2016) and during the intervention (2017-2019).

## Data Availability

The food composition data used in this study is from FoodSwitch. Any requests to access the data can be directed to foodswitch@georgeinstitute.org.au, however restrictions to the availability of these data may apply.

## Abbreviations

VSRP: Victorian Salt Reduction Partnership
UK: United Kingdom
