# Supplementary material for: Changes in sodium levels in Australian packaged foods between 2014 and 2019: an interrupted time series analysis of the impact of the Victorian Salt Reduction Partnership’s media advocacy strategy
Source: Int J Behav Nutr Phys Act. 2023 Jun 14;20:71. doi: 10.1186/s12966-023-01475-5 (PMC10268386; doi:10.1186/s12966-023-01475-5)
Supplement: Supplementary file 1 — Additional file 1: Supplementary Table 1. Estimated change in sodium levels in the targeted food products before and during the intervention. Supplementary Table 2. The number of products, mean sodium content (mg/100g) and standard deviation for each food category before (2014-2016) and during the intervention (2017-2019). [file 12966_2023_1475_MOESM1_ESM.docx]

**Supplementary Table 1. Estimated change in sodium levels in the targeted food products before and during the intervention**

| **Food category** | **Pre-intervention slope (2014-16) (95% CI)** | **Shift or change in the level of outcome prior to the intervention (between 2016 and 2017) (95% CI)** | **Intervention slope (2017-19) (95% CI)** | **Difference** **between pre-intervention and intervention slopes (95% CI)** |
| --- | --- | --- | --- | --- |
| **BREAD** |  |  |  |  |
| Leavened bread | **-7.84** (-12.27 to -3.41) | **3.53** (-7.72 to 14.77) | **-2.96** (-7.61 to 1.70) | **4.88** (-1.63 to 11.40) |
| Flat bread | **-6.88** (-14.68 to 0.93) | **-6.44** (-25.72 to 12.83) | **13.58** (5.29 to 21.87) | **20.46** (9.11 to 31.81) |
| Other bread | **-2.91** (-8.24 to 2.42) | **-4.49** (-17.36 to 8.39) | **-0.93** (-6.36 to 4.49) | **1.98** (-5.66 to 9.61) |
| **COOKING SAUCES** |  |  |  |  |
| Meal-based sauces | **-0.88** (-29.99 to 28.24) | **-18.14** (-80.38 to 44.10) | **-3.72** (-30.08 to 22.65) | **-2.84** (-42.64 to 36.96) |
| Pasta sauces | **-5.48** (-13.64 to 2.69) | **16.65** (-2.67 to 35.96) | **-17.28** (-25.66 to -8.89) | **-11.80** (-23.72 to 0.12) |
| Tomato paste | **-17.95** (-42.89 to 6.98) | **21.90** (-47.59 to 91.39) | **-23.70** (-51.82 to 4.43) | **-5.74** (-43.67 to 32.18) |
| **READY MEALS** |  |  |  |  |
| Ambient ready meals | **0.42** (-4.94 to 5.79) | **10.62** (-3.70 to 24.93) | **-1.64** (-8.22 to 4.93) | **-2.07** (-10.71 to 6.57) |
| Chilled ready meals | **-4.34** (-13.13 to 4.45) | **15.52** (0.17 to 30.87) | **-10.32** (-17.28 to -3.36) | **-5.98** (-17.45 to 5.50) |
| Frozen ready meals | **2.56** (-5.97 to 11.08) | **21.16** (0.98 to 41.34) | **-10.91** (-19.10 to -2.72) | **-13.47** (-25.40 to -1.53) |
| **DIPS AND CRACKERS** |  |  |  |  |
| Dips | **1.81** (-5.15 to 8.76) | **9.15** (-7.58 to 25.88) | **-2.57** (-9.75 to 4.61) | **-4.37** (-14.42 to 5.67) |
| Plain dry biscuits | **-26.75** (-39.16 to -14.34) | **11.70** (-20.21 to 43.61) | **-2.22** (-16.03 to 11.59) | **24.53** (5.87 to 43.19) |
| Savoury biscuits | **-27.38** (-98.38 to 43.63) | **-102.09** (-274.08 to 69.90) | **56.13** (-10.30 to 122.56) | **83.51** (-13.85 to 180.87) |
| **PROCESSED MEAT** |  |  |  |  |
| Bacon | **-35.74** (-64.80 to -6.67) | **-13.39** (-77.11 to 50.33) | **8.80** (-16.02 to 33.62) | **44.54** (6.36 to 82.72) |
| Sausages | **8.09** (-7.28 to 23.47) | **42.22** (11.02 to 73.43) | **-6.32** (-21.03 to 8.40) | **-14.41** (-36.05 to 7.23) |
| Sliced meat | **-4.95** (-20.12 to 10.23) | **6.45** (-35.96 to 48.85) | **12.53** (-4.36 to 29.41) | **17.47** (-5.29 to 40.23) |
| Ham | **1.52** (-30.47 to 33.50) | **73.08** (-9.20 to 155.36) | **-26.72** (-60.69 to 7.25) | **-28.24** (-75.28 to 18.80) |
| **ASIAN-STYLE SAUCES** |  |  |  |  |
| Asian-style sauces | **18.81** (-50.78 to 88.41) | **-13.22** (-175.24 to 148.79) | **75.98** (10.63 to 141.32) | **57.16** (-38.94 to 153.26) |

Note: Beta-estimates are in mg/100g. A significant difference indicates a treatment effect over time. Green represents decrease in sodium levels. Red represents increase in sodium levels.

**Supplementary Table 2. The number of products, mean sodium content (mg/100g) and standard deviation for each food category before (2014-2016) and during the intervention (2017-2019)**

| **Food category** | **2014** | **2015** | **2016** | **2017** | **2018** | **2019** |
| --- | --- | --- | --- | --- | --- | --- |
| **BREAD** | | | | | | |
| Leavened bread | 181; 422 (95) | 171; 418 (86) | 138; 395 (85) | 160; 406 (80) | 255; 410 (106) | 217; 403 (90) |
| Flat bread | 110; 551 (231) | 88; 543 (232) | 92; 565 (232) | 94; 583 (221) | 119; 548 (227) | 108; 540 (256) |
| Other bread | 173; 427 (134) | 136; 445 (122) | 137; 409 (114) | 150; 404 (130) | 197; 409 (122) | 178; 409 (136) |
| **COOKING SAUCES** | | | | | | |
| Meal-based sauces | 362; 1890 (1928) | 315; 1841 (1838) | 330; 1690 (1879) | 362; 1816 (1977) | 351; 1752 (1814) | 321; 1737 (1957) |
| Pasta sauces | 216; 457 (298) | 211; 439 (260) | 224; 415 (256) | 195; 424 (262) | 211; 402 (250) | 224; 374 (237) |
| Tomato paste | 22; 416 (336) | 18; 391 (336) | 19; 344 (292) | 14; 373 (287) | 18; 342 (277) | 18; 331 (270) |
| **READY MEALS** | | | | | | |
| Ambient ready meals | 124; 357 (189) | 97; 406 (583) | 116; 419 (557) | 101; 350 (163) | 85; 408 (622) | 73; 456 (692) |
| Chilled ready meals | 94; 314 (112) | 86; 305 (95) | 129; 281 (92) | 130; 284 (92) | 235; 306 (216) | 209; 258 (169) |
| Frozen ready meals | 279; 306 (125) | 239; 328 (171) | 267; 324 (207) | 257; 328 (209) | 345; 327 (185) | 317; 322 (142) |
| **DIPS AND CRACKERS** | | | | | | |
| Dips | 264; 473 (196) | 255; 478 (204) | 222; 483 (182) | 282; 497 (242) | 292; 509 (306) | 254; 519 (314) |
| Plain dry biscuits | 180; 591 (303) | 168; 549 (243) | 179; 534 (243) | 166; 532 (272) | 171; 510 (250) | 174; 532 (305) |
| Savoury biscuits | 171; 770 (312) | 180; 736 (296) | 161; 721 (292) | 179; 697 (314) | 207; 664 (300) | 204; 804 (1417) |
| **PROCESSED MEAT** | | | | | | |
| Bacon | 44; 1224 (336) | 45; 1161 (348) | 37; 1088 (191) | 51; 1046 (179) | 65; 1087 (160) | 64; 1066 (83) |
| Sausages | 70; 660 (174) | 51; 692 (197) | 69; 636 (158) | 73; 711 (242) | 78; 670 (205) | 69; 681 (168) |
| Sliced meat | 64; 773 (263) | 50; 732 (239) | 47; 805 (277) | 39; 842 (261) | 49; 897 (265) | 48; 940 (327) |
| Ham | 70; 1113 (481) | 76; 1021 (269) | 72; 1088 (249) | 62; 1081 (227) | 70; 1065 (212) | 70; 1013 (221) |
| **ASIAN-STYLE SAUCES** | | | | | | |
| Asian-style sauces | 43; 5501 (2103) | 45; 5556 (2140) | 48; 5815 (2306) | 51; 5810 (2448) | 49; 5827 (2429) | 47; 5974 (2342) |
